# Insomnia, Benzodiazepine Use, and Falls among Residents in Long-term Care Facilities

**DOI:** 10.3390/ijerph16234623

**Published:** 2019-11-21

**Authors:** Yu Jiang, Qinghua Xia, Jie Wang, Peng Zhou, Shuo Jiang, Vinod K. Diwan, Biao Xu

**Affiliations:** 1 Department of Epidemiology, School of Public Health, Fudan University, Shanghai 200032, China; jiangyukx@126.com; 2 Changning District Centre for Disease Control and Prevention, Shanghai 200052, China; xiaqinghua56@126.com (Q.X.); wangjiecat@163.com (J.W.); juju7881@126.com (P.Z.); 14211020057@fudan.edu.cn (S.J.); 3 Key Lab of Health Technology Assessment, National Health Commission of the People’s Republic of China (Fudan University), Shanghai 200032, China; 4 Department of Public Health Sciences (Global Health/IHCAR), Karolinska Institute, 17177 Stockholm, Sweden; vinod.diwan@ki.se

**Keywords:** falls, long-term care, sleep quality, insomnia, benzodiazepines

## Abstract

*Background:* Falls are leading cause of injury among older people, especially for those living in long-term care facilities (LTCFs). Very few studies have assessed the effect of sleep quality and hypnotics use on falls, especially in Chinese LTCFs. The study aimed to examine the association between sleep quality, hypnotics use, and falls in institutionalized older people. *Methods:* We recruited 605 residents from 25 LTCFs in central Shanghai and conducted a baseline survey for sleep quality and hypnotics use, as well as a one-year follow-up survey for falls and injurious falls. Logistic regression models were applied in univariate and multivariate analysis. *Results:* Among the 605 participants (70.41% women, mean age 84.33 ± 6.90 years), the one-year incidence of falls and injurious falls was 21.82% and 15.21%, respectively. Insomnia (19.83%) and hypnotics use (14.21%) were prevalent. After adjusting for potential confounders, we found that insomnia was significantly associated with an increased risk of falls (adjusted risk ratio (RR): 1.787, 95% CI, 1.106–2.877) and the use of benzodiazepines significantly increased the risk of injurious falls (RR: 3.128, 95% CI, 1.541–6.350). *Conclusion:* In elderly LTCF residents, both insomnia and benzodiazepine use are associated with an increased risk of falls and injuries. Adopting non-pharmacological approaches to improve sleep quality, taking safer hypnotics, or strengthening supervision on benzodiazepine users may be useful in fall prevention.

## 1. Introduction

By the end of 2017, China’s population over the age of 65 years old accounted for 11.4% of the total population, while it reached 20.6% in Shanghai. About 3% of Shanghai’s elderly people live in long-term care facilities (LTCFs), and the proportion will continue to rise due to the aging population and the weakening care function of families. The rate of falls in LTCFs in developed countries was 43% on average or 1.6 falls/bed [1,2]. In China, few studies have measured the frequency of falls in LTCFs with a wide range of estimation from 13% to 39% [3,4]. As the top safety issue for aged care settings in China, falls not only pose a major health risk among the residents, but also pose a threat to the long-term care industry. The identification of modifiable risk factors and action to eliminate them have been proven an effective way to reduce the occurrence of falls.

Sleep disturbances, although common in older people, are even more prevalent and severe in institutional older adults than their community-dwelling counterparts [5] due to higher burden of comorbidities, decreased physical activities, less social interaction, less daylight exposure, more environmental noise and nocturnal care practices [6]. The prevalence of poor sleep quality among Chinese long-term care residents ranged from 33% to 73% [7] and the prevalence of insomnia was 24% (range 13%–30%) in seven European countries and Israel [8]. Poor sleep quality can lead to many adverse health effects like reduced balance [9], cognitive impairment [10] and even increased mortality [11]. It also has been associated with an increased risk of falls, but the results in the literature are mixed [12]. 

Additionally, sleep aid medicines are also reported to increase the risk of falling. A meta-analysis found that sedatives and hypnotics increased the likelihood of falls by factor of 47%, and so did benzodiazepines, by 57% in older adults [13]. Another systematic review and meta-analysis reported that both benzodiazepines and Z-drug use were significantly associated with an increased risk of hip fracture [14]. The co-existence of poor sleep quality and hypnotics use makes it difficult to tell whether they have independent effect on falls. Only a few studies took both insomnia and hypnotics use into account at the same time. A few studies that accounted for both factors have generated conflicting findings. One study among 34,163 elderly nursing home residents suggested that untreated insomnia independently predicted falls and hypnotics use may have protective effects on falls [15]. Another study conducted among 9843 community-dwelling older adults concluded that sleep problems and sleep medication use are key risk factors for falls and sleep medicines did not have a protective effect on falls [16]. The objective of this study is to examine the association of sleep quality and hypnotic use with the risk of falls, using a prospective cohort study in institutionalized residents, to provide a basis for designing effective fall-prevention strategies in this population.

## 2. Materials and Methods 

### 2.1. Settings and Participants

A two-stage sampling was applied to recruit study subjects. The first stage was to choose the LTCFs. Based on the size and ownership of LTCFs in Shanghai, 25 LTCFs were randomly selected from 38 LTCFs in Changning district, which has a good geographical representation of the Shanghai central area. Among the sampled LTCFs, four were public and 21 were private. The second stage was to identify study subjects from the participating LTCFs. The inclusion criteria were: (1) able to ambulate independently; (2) can communicate normally; (3) with expected survival time of more than 1 year; and (4) are willing to participate in the survey. Residents who met the criteria were invited by doctors and administrators who were familiar with the health status of the elderly. Among the 3858 elderly people residing in the LTCFs, 739 were eligible and completed the baseline survey, of which 605 completed a one-year follow-up survey and 134 (18.1%) were lost to follow-up. Of the 134 residents, 69 died (51.5%) and 65 (48.5%) were transferred or discharged from the facilities.

### 2.2. Determination of Falls

A fall was defined as unintentional rest on the ground or lower place [17]. All participating residents were followed up with for 1 year after completing the baseline survey. During the period, information on the occurrence of falls was collected from three types of records (care records, accident records and medical records) and by interviewing the residents.

### 2.3. Sleep Quality and Medication Use

Sleep quality was assessed using the Athens Insomnia Scale (AIS) [18] at baseline. It contained eight items, where each with a rating scale of 0-3, and a total score ranged from 0 to 24. An AIS score ≥6 indicated insomnia (previous month). The information of sedative sleeping pills used within the preceding two weeks was obtained by excerpting the records of the facilities or by reviewing the pill bottles of the participants. Among the hypnotics, benzodiazepines included diazepam, flurazepam, oxazepam, clonazepam, chlordiazepam, alprazolam, estazolam, triazolam, and midazolam etc., and non-benzodiazepines included zolpidem, eszopiclone, zaleplon, and traditional Chinese medicines. A hypnotic used off label for another purpose was also regarded as a hypnotic.

### 2.4. Potential Confounders

The survey was conducted using questionnaires designed by the study administrators. The demographics and health information were extracted by reviewing the health records, and other information was collected through face-to-face interviews. The care level of residents (level 1, 2 and 3) was assessed by physicians according to Shanghai Facilities and Service Requirements of Elderly Institutions. Residents in care level 3 were less frail and required minimum basic care.

Cognitive ability was assessed using the Mine-Mental State Examination Scale (MMSE) [19]. A total score of less than 27 was regarded as cognition decline. Depression was assessed by using the Geriatric Depression Scale (GDS) [20]. A total score of more than 10 points was considered as having depression symptoms. Balance ability was measured on-site by trained community doctors using the X-16 Balance Ability Test Scale. The maximum total score was 24 [21], and a score of less than 17 was considered as balance decline. 

### 2.5. Ethical Consideration

The project was approved by the Institutional Review Board (IRB) of the Changning Centre for Disease Control and Prevention, and written informed consent was obtained from all interviewed subjects.

### 2.6. Statistical Analysis

EpiData 3.0 (The EpiData Association, Odense, Denmark) was used for data entry, and SAS version 9.2 (SAS Institute, Inc., Cary, NC) was used for statistical analyses. The main independent variables were insomnia (yes/no) and use of hypnotics and benzodiazepines (yes/no) and the dependent variables were falls and fall-related injuries. The Charlson comorbidity index [22] was calculated, indicating the health status of the participants. Chi Square tests were performed to compare differences in categorical characteristics by insomnia and hypnotics use status. Univariate and multivariate logistic regression models were used to assess sleep quality and hypnotics use in relation to falls or injurious falls, with estimation of risk ratio (RR) and 95% confidence intervals (CIs). Covariates with a univariate statistical significance of p < 0.1 and those thought to be potential risk factors of falls in literature were included as potential confounders in the multivariate models. Collinearity among the covariates was assessed. Results were considered significant when *p* < 0.05.

## 3. Results

### 3.1. Basic Characteristics of the Participants

Among the 605 residents who completed both the baseline and follow-up surveys, 179 were male (29.59%), with an average age of 84.33(SD: 6.90) years old. The majority were 63.47% were aged 80–89 years. There were 206 (34.05%), 287 (47.44%), and 112 (18.51%) residents in the care level of 1 to 3, respectively. A total of 71.24% participants had comorbidity conditions, 64.63% suffered from mild or moderate cognition decline, and 63.97% had balance impairment. About thirty-five percent of the residents had depression symptoms (Table 1).

### 3.2. Sleep Quality, Hypnotics Use, and Sleep-Related Behaviors

According to AIS, 19.83% of residents had insomnia. Eighty-six residents (14.21%) took hypnotics, 43 of them took benzodiazepines, and 43 took non-benzodiazepines. Hypnotics use was reported among 37 (30.83%) insomniacs, of whom 19 took benzodiazepines and 18 used non- benzodiazepines.

About 20.66% and 10.91% of the residents had less than 6 h of sleep during nighttime and whole day period, respectively. Having a habit of napping happened in 76.69% of the residents and habitually visiting toilet for more than 3 times during nighttime happened in 14.38% participants (Table 1). 

Increasing comorbidity conditions, lack of nap during the daytime and more than three times nocturnal toilet visits were statistically significantly associated with increased likelihood of both insomnia and hypnotics use. Female, depression, less than 6 h of sleep duration during the night or whole day were significantly associated with insomnia only (see Table 1).

### 3.3. Sleep Quality, Hypnotics Use, and Falls

Of the 605 follow participants, 21.82% and 15.21% were reported to have fallen and suffered injurious falls during the 1-year follow-up period. Table 2 shows the unadjusted relationship of falls and injurious falls with sleep quality, hypnotics use, and potential confounders. In comparison to residents with no baseline insomnia, insomniacs had significantly higher incident falls (χ^2^ = 8.512, *p* = 0.004) and injurious falls (χ^2^ = 7.667, *p* = 0.006). Baseline hypnotics use was associated with a non-significant higher risk of falls (χ^2^ = 3.091, *p* = 0.079) but significantly greater risk of future injurious falls (χ^2^ = 5.037, *p* = 0.025). Benzodiazepines (χ^2^ = 4.633, *p* = 0.031) rather than non-benzodiazepines significantly increased the risk of falls and injurious falls.

In combined analyses, the incidence of falls and injurious falls was the lowest among residents who reported no insomnia and hypnotics use. The rate was followed by that in those who had no insomnia but took hypnotics, and then by insomniacs without taking hypnotics. Insomniacs who also took benzodiazepines at the same time had the highest incidence of falls and related injuries. Residents with insomnia had higher rate of falls regardless of hypnotics use status, while the elderly who took hypnotics had a higher incidence of falls regardless of sleep quality (Table 2).

Compared to those who did not take a nap, the elderly who took nap for less than 2 h had a lower incidence of falls, while the elderly who had longer than 2 h nap incurred a higher rate of falls, and the difference was statistically significant (χ^2^ = 6.302, *p* = 0.043). For injurious falls, a similar pattern was observed (χ^2^ = 7.759, *p* = 0.021). No statistically significant difference in incidence of falls and injurious falls was found between residents with different sleep durance and nighttime toilet visits (Table 2).

A logistic regression model was used to analyze the association of insomnia and benzodiazepines with incident falls or injurious falls. In an age adjusted model, both insomnia and benzodiazepine use significantly increased the risk of falls and injurious falls. After simultaneously putting age, insomnia and benzodiazepine use into the same model, benzodiazepines use did not significantly increase the risk of falls, while insomnia remained a significant predictor of falls, and both were risk factors for injurious falls. After introducing other potential confounders into the model, insomnia was still an independent risk factor for falls (Adjusted risk ratio (RR): 1.787; 95% CI, 1.106–2.877), while taking benzodiazepines was independent risk of injurious falls (RR: 3.128; 95% CI, 1.541–6.350) (Table 3).

## 4. Discussion

Sleep problems, hypnotic medicine use, and falls are common complaints in LTC settings. However, there were few studies focused on addressing sleep as a way to minimize falls in residents in LTCFs [23]. In China, sleep research in LTC residents is still at the beginning, and only a few cross-sectional studies and interventions have been reported [7]. The literature on the effect of sleep problems and hypnotics on falls is mixed, and there are only a few studies that considered both factors. This study used prospective cohort study design to analyze the independent effect of sleep problems and hypnotics use on falls and injurious falls in residents from 25 LTCFs in central Shanghai. To our knowledge, it is the first study of its kind in China. We found that insomnia was significantly associated with an increased risk of falls and the use of benzodiazepines significantly increased the risk of injurious falls, independent of potential confounders.

The annual incidence of falls and injurious falls among the subjects was 21.82% and 15.21%, higher than that among Chinese community-dwelling elderly adults [24] but lower than that in American and Australian older institutionalized residents [15,25]. AIS assessments indicated that about one in five residents suffered from insomnia. Among the participants, 14.21% took hypnotics (30.83% among insomniacs), 20.66% had less than 6 h of nighttime sleep. These results were roughly comparable with similar studies conducted in seven European countries, Israel [8] and Australia [25], adding evidence to the literature that poor sleep and hypnotics use were prevalent in LTCFs.

It has been implicated that poor sleep (sleep disturbance, short sleep durance, etc.) is associated with an elevated risk of fall among elderly adults both in community settings or long-term care settings [16,24,26,27,28]. A study of 34,163 elderly nursing home residents reported that it was insomnia (OR = 1.52; 95%CI = 1.38–1.66) rather than hypnotics use (OR = 1.03; 95%CI = 0.98–1.30) that predicted a significant greater risk of falls. Another LTCF based study (OR = 4.5; 95%CI = 1.9–12.2) [25] and a community-based study (OR = 1.36; 95%CI = 1.07–1.74) [28] also reported that poor sleep was an independent risk factor of falls after adjusting for confounding factors. In the current study, insomnia was significantly associated with falls in all models, although the effect estimates attenuated after adjusting for benzodiazepines use or other confounding variables. Insomniacs had a 78.7% higher greater risk for future falls (RR = 1.787, 95%CI = 1.106–2.887). However, as for injurious falls, after putting insomnia and other confounders into the model, we can only see a non-significant association (RR = 1.683, 95%CI = 0.977–2.899) between insomnia and injurious falls (refer to Table 3).

Hypnotics, commonly used medicines to improve sleep quality, are often associated with falls-related injuries. They can be divided into benzodiazepines and non-benzodiazepines (including “Z-drugs”). Benzodiazepines have long been associated with fractures [29,30] and injurious falls [31]. General practitioners believed that non-benzodiazepines are safer due to a shorter half-life so that the prescription of non-benzodiazepines has increased while that of benzodiazepines decreased over the past decade in some developed countries. However, some non-benzodiazepines was also found to be associated with an increased risk of fracture in insomnia patients [32]. Most of the prior studies failed to simultaneously consider sleep quality and hypnotics use. Two large-scale community studies analyzed the combined effect of sleep medication and sleep problems and demonstrated that sleep problems added to sleep medication use increase the risk of falls [16,33]. In this study, even in bivariate analyses, non-benzodiazepines use were not significantly associated with falls or injurious falls, so we only analyzed the effect of benzodiazepines in multivariate models. In combined bivariate analyses, benzodiazepine users with insomnia had the highest risk of falls and injurious falls compared to any other groups and benzodiazepines users without insomnia symptoms and insomniacs not taking benzodiazepines also had an elevated risk of falls and injurious falls. To our surprise, insomniacs with non-benzodiazepines use had the lowest incidence of injurious falls, indicating that non-benzodiazepines may have some protective effect with respect to injurious falls (refer to Table 2). The inclusion of traditional Chinese medicine in non-benzodiazepines may in part explain the results and the association warrants confirmation by future studies. In our study, after adjusting for potential confounders, baseline benzodiazepines use still predicted future injurious falls (RR = 3.128, 95%CI = 1.541–6.350) while only marginally associated with falls (RR = 1.891, 95%CI = 0.947–3.776) (refer to Table 3). 

Taken together, we could infer that both insomnia and benzodiazepines use may play a role in falls or injurious falls, with insomnia driving the association with falls while benzodiazepines make the major contribution to the association with injurious falls. The results indicated that treating insomnia may be benefits to reduce the risk of falls in LTCF residents, while prescription of benzodiazepines to elderly adults should be done with caution. Findings from this study are partly consistent with several similar studies, and the difference between our studies and previous studies may derive from the intrinsic differences between subjects or the different constitution of hypnotics. Further studies are needed to confirm these associations.

This study also found that comorbidity conditions, sleep durantion, daytime nap, and nocturnal toilet visits were associated with insomnia, which may be modifiable factors to improve sleep (refer to Table 1). Daytime nap habits were found to be associated with higher risk of both outcome variables in bivariate analyses, but the association did not exist in the multiple model. Its effect on falls may be explained by insomnia (refer to Table 2 and Table 3). Consistent with some previous studies [24,26], we did not find a significant relationship between sleep durantion and falls in this study (refer to Table 2).

Our study has its strengths and limitations. The prospective study design assures the exposures precede outcomes which is imperative in the causal inference. Having a relatively large sample size, taking both sleep quality and hypnotic medicine use into account, and adjusting for a variety of potential confounders improved the validity and reliability of the association between exposure and outcomes. However, some limitations should be noted. Insomnia was assessed based on self-reported information, which was a symptom rather than a diagnosis. AIS was applied in assessment, which was not used as widely as the Pittsburgh Sleep Quality Index (PSQI). These reduced the ability to compare findings from this study with some prior studies. Since the AIS score has been reported to be strongly correlated with the PSQI [34], AIS, which comprises only eight items that can be easily completed within a short period, may be an ideal tool to assess subjective insomnia and to even identify individuals at an increased risk of falls in LTCF residents. Validation studies of AIS against other standard measures such as PSQI and studies using actigraphy in assessing objective sleep quality are needed in the future. Due to the limited amount of subjects who took hypnotics in our study, we were unable to conduct further subgroup analyses based on specific types of benzodiazepines and non-benzodiazepines. Non-benzodiazepines contained traditional Chinese medicines, which may dilute the effect of non-benzodiazepines to a certain extent. The effect of specific drugs such as traditional Chinese medicine and long-acting benzodiazepines on falls is worthy of further investigation. The research participants were selected based on the ability of walking and communication, and was thus not a representative sample of all residents in LTCFs, so the results can only be extrapolated to the relatively independent residents in LTCFs. Residents with visual disability, severe hearing loss, and inability to communicate, who had been excluded from this study, may have a greater risk of falling and are worth equal attention. We also cannot eliminate the possibility that some confounding factors were not measured and included in the analysis, affecting the true relationship between sleep quality and falls. We cannot exclude the possibility that sleep patterns could have changed during the follow-up period, and may not accurately reflect the participants’ sleep at the time falls occurred. As indicated by some studies, sleep disturbances among LTC residents can be persistent for up to 12 months [35]. The change in sleep pattern may not affect the association to a large extent.

## 5. Conclusions

In conclusion, both insomnia and benzodiazepine use, are associated with falls and injurious falls among LTC residents, where insomnia being a major risk factor for falls and benzodiazepine use for injurious falls. Findings from this study shed light on practices on fall prevention in LTC settings. To prevent falls in LTCFs, improving sleep quality should be prioritized with non-pharmacological measures such as napping monitoring, supports for urination at night, and protective environments for avoiding falls, etc. For those who are administered hypnotics, additional monitoring and people-centered individual care should be guaranteed. 

## Figures and Tables

**Table 1:** Characteristics of the study participants by sleep quality and hypnotics use (*n* = 605).

Characteristics	Total (%)	Insomnia (*n* = 120)	Non-Insomnia (*n* = 485)	*p*-Value *	Hypnotic User (*n* = 86)	Non-Hypnotic User (*n* = 519)	*p*-Value *
Gender							
Male	179 (29.59)	12.85	87.15	0.005	12.85	87.15	0.533
Female	426 (70.41)	22.77	77.23		14.79	85.21	
Age							
<80 years	100 (16.53)	21.00	79.00	0.091	12.00	88.00	0.564
80–89 years	384 (63.47)	17.45	82.55		15.36	84.64	
≥90 years	121 (20.00)	26.45	73.55		12.40	87.60	
Care level							
Level 1	206 (34.05)	19.42	80.58	0.602	15.05	84.95	0.501
Level 2	287 (47.44)	18.82	81.18		14.98	85.02	
Level 3	112(18.51)	23.21	76.79		10.71	89.29	
Charlson Comorbidity Index							
0	174 (28.76)	16.09	83.91	<0.001	8.62	91.38	<0.001
1–2	208 (34.38)	13.46	86.54		10.58	89.42	
≥2	223 (36.86)	28.70	71.30		21.97	78.03	
Cognitive state							
Intact	214 (35.37)	21.03	78.97	0.586	16.36	83.64	0.265
Decline	391 (64.63)	19.18	80.82		13.04	86.96	
Depression							
No	399 (65.95)	13.78	86.22	<0.001	13.53	86.47	0.504
Yes	206 (34.05)	31.55	68.45		15.53	84.47	
Balance ability							
Intact	218 (36.03)	21.10	78.90	0.558	14.22	85.78	0.998
Decline	387 (63.97)	19.12	80.88		14.21	85.79	
Nighttime sleep time							
<6 h	125 (20.66)	48.00	52.00	<0.001	19.20	80.80	0.073
≥6 h	480 (79.34)	12.50	87.50		12.92	87.08	
Total sleep time							
<6 h	66 (10.91)	63.64	36.36	<0.001	21.21	78.79	0.085
≥6 h	539 (89.09)	14.47	85.53		13.36	86.64	
Nap in daytime							
None	141 (23.31)	26.24	73.76	0.048	17.73	82.27	0.013
<2 h	375 (61.98)	18.93	81.07		15.20	84.80	
≥2 h	89 (14.71)	13.48	86.52		4.49	95.51	
Nighttime toilet visits							
<3 times	518 (85.62)	16.99	83.01	<0.001	12.55	87.45	0.004
≥3 times	87 (14.38)	36.78	63.22		24.14	75.86	

* *p*-values were obtained from the Chi-square tests.

**Table 2:** Unadjusted relationship of sleep quality and hypnotics use with falls or injurious falls in the study participants (*n* = 605).

Sleep and Hypnotics Use	Fallers (*n* = 132)	Non-Fallers (*n* = 473)	*p*-Value *	Injurious Fallers (*n* = 92)	Non-Injurious Fallers (*n* = 513)	*p*-Value *
Sleep quality *						
Insomnia	31.67	68.33	0.004	23.33	76.67	0.006
Non-insomnia	19.38	80.62		13.20	86.80	
Hypnotics use						
Yes	29.07	70.93	0.079	23.26	76.74	0.025 *
No	20.62	79.38		13.87	86.13	
Benzodiazepines use						
Yes	34.88	65.12	0.031	34.88	65.12	<0.001
No	20.82	79.18		13.70	86.30	
Non-benzodiazepines use						
Yes	23.26	76.74	0.813	15.48	84.52	0.499
No	21.71	78.29		11.63	88.37	
Sleep quality & hypnotics use						
No insomnia, no hypnotics use	18.81	81.19	0.044	12.16	87.84	0.003
No insomnia, hypnotics use	24.49	75.51		22.45	77.55	
Insomnia, no hypnotics use	30.12	69.88		22.89	77.11	
Insomnia, benzodiazepines use	36.84	63.16		36.84	63.16	
Insomnia, non-benzodiazepines use	33.33	66.67		11.11	88.89	
Nighttime sleep durance						
<6 h	24.24	75.76	0.507	17.60	82.40	0.403
≥6 h	21.52	78.48		14.58	85.42	
Total sleep durance						
<6 h	24.00	76.00	0.613	19.70	80.30	0.282
≥6 h	21.25	78.75		14.66	85.34	
Nap in daytime						
None	24.31	75.69	0.043	19.44	80.56	0.021
<2 h	18.37	81.63		11.66	88.34	
≥2 h	28.81	71.19		20.34	79.66	
Nighttime toilet visits						
<3 times	21.04	78.96	0.260	14.67	85.33	0.371
≥3 times	26.44	73.56		18.39	81.61	

* *p*-values were obtained from the Chi-square tests.

**Table 3:** Adjusted association of insomnia and hypnotics use with falls and injurious falls in the study participants (*n* = 605).

Model	Predictive Factor	Falls			Injurious Falls		
		RR *	95% CI	*p*-Value	RR *	95% CI	*p*-Value
Model1	age	0.994	(0.967–1.022)	0.687	1.009	(0.976–1.043)	0.613
	Insomnia	1.934	(1.238–3.021)	0.004	1.994	(1.211–3.282)	0.007
Model2	age	0.995	(0.967–1.023)	0.710	1.008	(0.975–1.043)	0.627
	Benzodiazepines	2.047	(1.058–3.960)	0.033	3.355	(1.713–6.570)	0.0004
Model3	age	0.994	(0.966–1.022)	0.662	1.008	(0.974–1.042)	0.652
	Insomnia	1.815	(1.152–2.860)	0.010	1.749	(1.046–2.923)	0.033
	Benzodiazepines use	1.755	(0.892–3.451)	0.103	2.919	(1.467–5.808)	0.002
Model4	Insomnia	1.787	(1.106–2.887)	0.018	1.683	(0.977–2.899)	0.061
	Benzodiazepines use	1.891	(0.947–3.776)	0.071	3.128	(1.541–6.350)	0.002
	age	0.993	(0.964–1.023)	0.662	1.009	(0.974–1.046)	0.609
	female	0.868	(0.560-1.347)	0.529	1.039	(0.618–1.746)	0.885
	Care Level 1	Ref	Ref	Ref	Ref	Ref	Ref
	Care level 2	0.710	(0.457–1.103)	0.194	1.030	(0.612–1.733)	0.801
	Care Level 3	0.869	(0.490–1.541)	0.908	1.197	(0.615–2.332)	0.584
	Charlson comorbidity score	1.023	(0.884–1.185)	0.760	0.926	(0.776–1.105)	0.394
	Depression	1.014	(0.294–3.503)	0.982	1.818	(0.504–6.551)	0.361
	Nighttime toilet visits ≥3 times	1.135	(0.658–1.955)	0.650	1.106	(0.592–2.067)	0.752
	Don’t take nap	1.250	(0.782–2.000)	0.971	1.572	(0.928–2.663)	0.491
	Take nap less than <2 h	Ref	Ref	Ref	Ref	Ref	Ref
	Take nap ≥2 h	1.535	(0.884–2.667)	0.254	1.695	(0.893–3.215)	0.342

Note: RR refers to adjusted risk ratio.
